# A qualitative study of senior management perspectives on the leadership skills required in regional and rural Australian residential aged care facilities

**DOI:** 10.1186/s12913-022-08049-4

**Published:** 2022-05-18

**Authors:** Nathan Dawes, Stephanie M. Topp

**Affiliations:** Division of Tropical Health and Medicine, College of Public Health, Medical and Veterinary Sciences, James Cook University, I Building 41 I Room 114, 1 James Cook Drive Townsville QLD, 4811 Douglas, Australia

**Keywords:** Leadership, Management, Skills, Quality of care, Residential aged care

## Abstract

**Background:**

With increasing recognition of the quality and safety issues in residential aged care, there is an urgent need to better understand what skills senior managers require to deliver on the spectrum of leadership functions in residential aged care facilities. This qualitative study sought to explore the leadership skills that positively influence the quality of care within Australian residential aged care facilities and better understand the professional development needs of senior managers to positively influence care within these complex environments.

**Methods:**

We conducted semi-structured interviews with 19 senior managers purposively recruited from 14 high-performing non-government residential aged care facilities of varying geographical remoteness in northern Queensland, Australia. Participants held a range of professional roles, including Chief Executive Officer, Director of Nursing and Facility Manager, and had various professional qualifications. We used inductive thematic analysis to identify and categorise senior managers’ perspectives on the leadership skills and related strategies to promote quality of care.

**Results:**

Senior managers reported leadership skills in five major domains: i) communication and relationship management, ii) stewardship, iii) professional development, iv) health care knowledge and v) information technology and finance. Most participants highlighted communication and relationship management skills and responding to regulatory change as influential to residential aged care quality performance. Participants with different professional backgrounds often emphasised different skills.

**Conclusions:**

Participants identified a broad range of skills and strategies required by senior managers in Australian residential aged care facilities. Identifying different skills by differently trained individuals suggests more work is needed to understand and develop sector-specific professional development approaches to better prepare individuals to lead in this complex service environment.

**Supplementary Information:**

The online version contains supplementary material available at 10.1186/s12913-022-08049-4.

## Background

Compared with other nations, Australians are living longer than expected [1]. By 2057, it is projected there will be 8.8 million older people in Australia (22% of the population), and by 2097 approximately 25% of the population will be aged 65 years or over [2]. With extended longevity often accompanied by increasing health issues, population ageing is expected to increase demand for residential aged care [3].

As in many countries, residential aged care organisations in Australia face increasing service demand that runs concurrent with concerns about financial viability, workforce shortages, and associated quality of care [4, 5]. Recently, shortcomings of some aged care organisations and multiple incidences of substandard care were made public as part of a Royal Commission into Aged Care Quality and Safety [6]. The report highlighted that Australia’s residential aged care sector faces many structural challenges. First, the national aged care funding instrument is regarded as a “flawed” model, resulting in chronic underfunding and financial hardship for a majority of aged care organisations [6]. In the 2021/22 financial year, over half of the Australian residential aged care facilities recorded an operating loss [7]. This situation influences facilities’ capacity to recruit and retain sufficient and appropriately skilled staff [6]. Research pre-dating the Royal Commission has also demonstrated how these widespread resourcing challenges were exacerbated in rural and regional areas where competition with better paid ‘mainstream’ health services (e.g. hospital and family medicine practices) make it difficult for residential aged care facilities to recruit from a limited pool of skilled providers [8]. Resourcing constraints also limit the ability to access or update information technologies, subsequently reducing reporting accuracy and access to accredited virtual training opportunities [9].

In addition to these significant structural challenges to residential aged care organisations in Australia, the Royal Commission report also noted that leadership skills and strategies employed by residential aged care senior managers were lacking compared to other Australian mainstream health care organisations and international aged care services [10]. Effective leadership, together with a manager’s ability to provide strategic direction, is regarded by Anderson, Issel and McDaniel (2013) [11] as important in promoting quality in healthcare settings. Previous work has demonstrated how leadership is a process, that entails influence within a group setting or context, and involves achieving goals that reflect a common vision [12]. Empirical research in healthcare settings has also demonstrated that the personal attributes of leaders are linked to increased quality of care via their effects on employee job satisfaction and patient engagement [13], and, specific to aged care, through the empowerment of older persons to make informed decisions regarding their own care [10].

While the leadership of residential aged care services may be viewed through multiple lens [14], a skills perspective has often been employed to identify and describe the skills, knowledge and personal qualities required by managers to promote quality performance in healthcare settings [15, 16]. Overall, leadership competencies can be understood as the cumulative leadership skills, knowledge and personal qualities that have the potential to influence quality of care [16]. Although substantial research has been conducted around leadership skills in mainstream healthcare organisations [15, 17], comparatively little is known about the leadership skills or combinations of skills required in the distinctive settings of residential aged care facilities [18, 19]. In examining the issues and the progress made in leadership relevant to the residential aged care workforce, Jeon (2010) reported leadership and management promoted staff job satisfaction and employee retention, two factors linked to high-quality care. Research has also demonstrated the link between leadership styles and factors influencing quality of care, including staffing levels and workforce culture in residential aged care [20]. However, a majority of research exploring the role of leadership in promoting the quality of residential aged care has been conducted in the United States of America. None focuses explicitly on senior management skills in the Australian residential aged care sector [21].

This knowledge gap is notable because although mainstream healthcare organisations and residential aged care facilities share some common features, they also differ in a number of critical ways relevant to their leadership profile [22]. Clients’ purpose for attending and length of stay, the nature of clinical services delivered, the attendant organisational structures and staff skills mix required [21], and the broader financial and regulatory context differ in residential aged care compared with mainstream hospital settings [2]. Attending to these factors, the role, skills and personal qualities required by residential aged care senior managers are therefore potentially unique compared with other health settings. For example, senior managers of residential aged care facilities may be responsible for clinical care responsibilities but also and concurrently for institutional governance and risk operations, finance and asset performance, ethical conduct issues, people development, inter-professional collaboration and a range of commercial and political acumen [22]. Indeed residential aged care senior managers may require different types and combinations of leadership skills to achieve high-quality service outcomes as compared to their mainstream healthcare organisation counterparts [23].

Against the backdrop of an aging Australian population, the observation of limited leadership skills in residential aged care facilities by the Royal Commission, and considering the scarce empirical research conducted on leadership skills in this setting, there is a need for a closer examination of the leadership skill requirements of senior management in Australian residential aged care facilities. The current study aimed to qualitatively explore senior managers’ perspectives about which leadership skills are critical to providing high-quality care. In doing so, we aim to build the evidence base and better characterise and support this critical leadership group’s future professional development needs.

## Methods

### Study design

This study was exploratory, as with a few notable exceptions, there is little empirical evidence regarding how leadership does or should influence the quality of Australian residential aged care services [1, 19]. Qualitative methods were deemed appropriate to capture senior managers’ expressed beliefs, values, feelings, and motivations regarding important leadership skills required in residential aged care facilities [18]. We conducted in-depth interviews (IDIs) to gain a deeper understanding of participants’ views and experiences regarding the leadership competencies that influence quality of care in residential aged care facilities (Additional file 1).

### Study setting

**Fig. 1:**
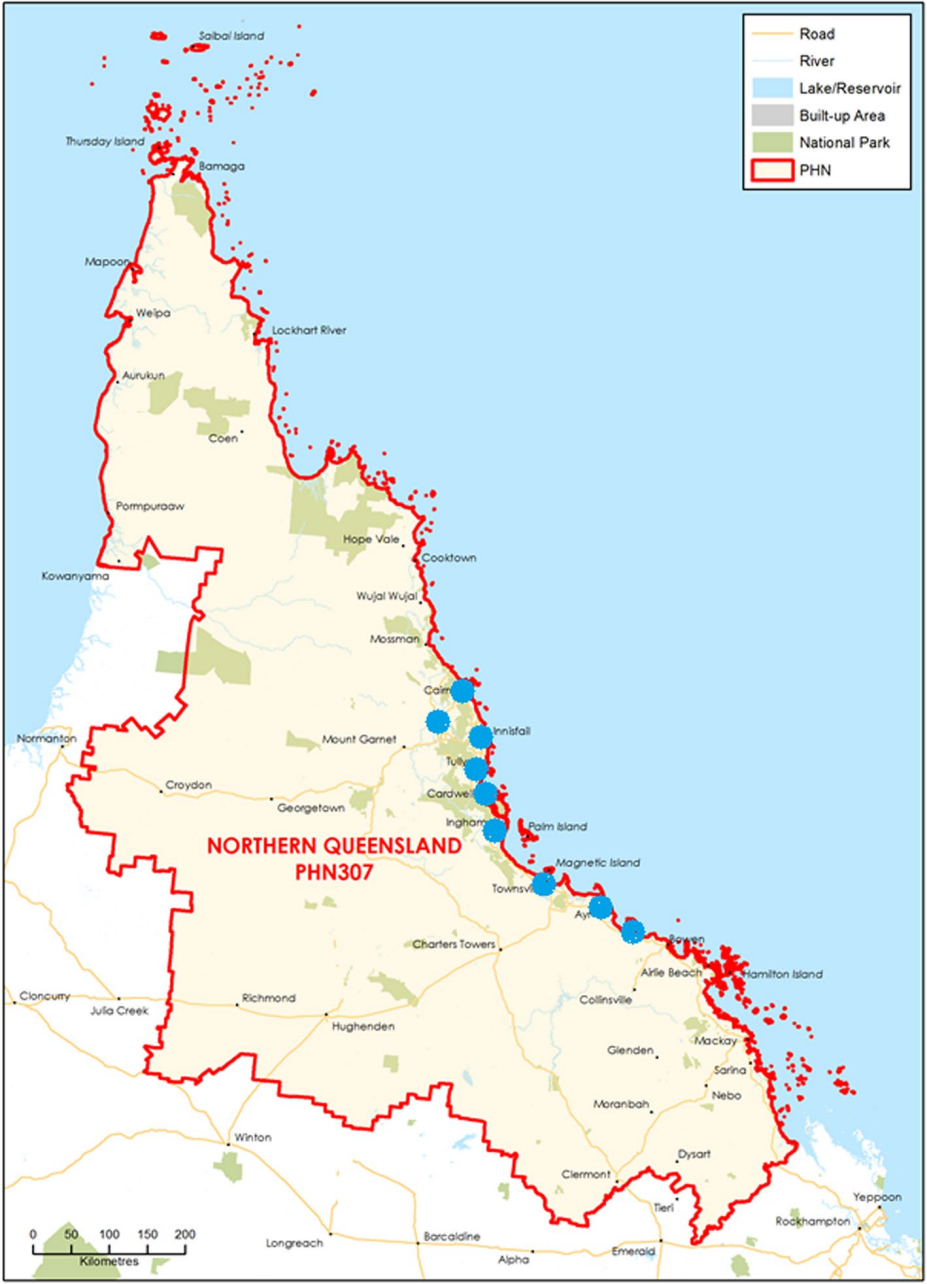
Northern Queensland Primary Health Network Region [24] - Study locations

Participants were included from aged care facilities in the region covered by Northern Queensland Primary Health Network (NQPHN) [24] (Fig. 1). Spanning an area of 510,000 km2, approximately twice the land size of the United Kingdom (UK), this tropical environment is home to approximately 700,000 people [24]. Most of the population is located within the regional centres of Cairns, Townsville, and Mackay, while approximately 8% of inhabitants live in remote and very remote areas. The Australian Statistical Geography Standard (ASGS) distinguishes five classes of relative remoteness across Australia (Australian Bureau of Statistics, 2019) [25]. The NQPHN region contains various degrees of geographical remoteness, including inner and outer regional, remote and very remote localities (ASGS 2 – 5). Facilities eligible for recruitment were in ASGS categories 2 – 5 and included for-profit and not-for-profit organisations.

### Site selection and participant recruitment

Site selection was conducted using GEN Aged Care Data [26] to obtain a list of ‘high performing’, non-government residential aged care facilities within the NQPHN region. ‘High performing’ residential aged care facilities obtained the maximum score (44/44) against the Accreditation standards and as assessed by the Aged Care Quality and Safety Commission in 2019. Targeting of ‘high performing’ facilities was taken to ensure a ‘strengths based’ approach to understanding how and in what ways leadership by senior management was positively influencing quality of care.

From the final 14 selected sites, a purposive sampling approach was used to select and recruit individual participants. Purposive sampling took account of participants’ current role and relevant experience within the sector to ensure critical reflection on the link between senior manager leadership skills and quality residential aged care in northern Queensland. Selection was designed to achieve a spread of roles (e.g. CEO, Director of Nursing, other administrative leadership roles).

Recruitment was conducted using a combination of email with phone follow-ups. Overall, 19 in-depth interviews (Table 1) were conducted face to face or over the phone with residential aged care managers between December 2019 and January 2020. All participants agreed to the interview being audio-recorded, transcribed and were provided with a copy of the transcription as an opportunity to correct or remove data before the analysis.

**Table 1:** Description of participants based on professional role, qualifications and ASRG category

Participant	Professional Role	Qualification/s	ASRG Category
1	CEO	Registered Nurse Diploma of Business & Human Resources	2
2	Facility Manager	Registered Nurse Management short course (over 10 years ago	2
3	CEO	Certificate in Business and Hospitality Financial cadetship	4
4	Clinical Care Coordinator	Registered Nurse	3
5	Senior Administration Officer	Certificate IV Administration	2
6	Director of Nursing	Registered Nurse	4
7	Residential Facility Manager	Registered Nurse Industry accreditation short courses	2
8	Facility Manager	Business short courses – no formal qualification reported	3
9	General Manager	No formal qualification	3
10	Director of Care	Emergency Nurse Bachelor of Geography and Social Policy	4
11	Clinical Care Manager	Registered Nurse	4
12	Clinical Care Manager	Registered Nurse	4
13	Clinical Care Manager	Registered Nurse Dip. Leadership and Management	2
14	Facility Manager	Dip. Management Bachelor of Business	2
15	Clinical Operations Manager	Registered Nurse	2
16	Facility Manager	Registered Nurse	4
17	Director of Nursing	Registered Nurse	2
18	Facility Manager	Bachelor of Hospitality	2
19	Facility Manager	Registered Nurse	5

### Data management and analysis

A range of approaches were conducted to ensure the rigour and credibility of the study findings. Handwritten memos were collated immediately after each interview to ensure a reflexive stance was maintained in relation to the research and participants. The data from each interview was transcribed verbatim into separate documents and then checked by the authors for accuracy against the original recording. ND assigned a unique identifier to each transcript denoting the service location: inner and outer regional, remote and remote very localities (ASGS 2 – 5); the participant’s managerial title; and the interview number for that service location and position title. Each participant was emailed a copy of the transcript for checking.

Thematic analysis was conducted using NVivo v12 software [27]. Open coding was performed, where codes were created based on identified topics and assigned to specific sections of transcripts [28]. Coding was guided by the study’s exploratory and involved giving text from across the dataset to a label [29].

## Results

### Overview

We present our findings of reported leadership skills under five inductively identified domains i) communication and relationship management, ii) stewardship, iii) professional development, iv) knowledge of the healthcare environment, and v) information technology (IT) and finance. Domains and the leadership skills that fall within them are referred to as ‘domains’ and ‘skills’, respectively to improve clarity. Participants were purposefully grouped into two categories to explore potential differences between the leadership skills described by those with a health qualification (*n* = 13) and those without a health qualification (*n* = 6).

### Communication and relationship management

‘Communication and relationship management’ skills included a leader’s ability to communicate clearly and concisely with internal and external stakeholders, share clinical and industry-related knowledge and employ effective complaint management processes within the organisation. Most study participants strongly emphasised relationship management and communication skills, however skills, including the ability to negotiate with external stakeholders, were less of a focus.

Participants with a formal health qualification reported that a manager’s ability to build and nurture collaborative relationships with residents, staff, and external providers was important to promote an organisation’s quality performance.

“So, when you are dealing with the various stakeholders, trying to be a bit more collaborative as opposed to directive. This can be an effective way to developing rapport and longstanding relationships.” Clinical Care Coordinator, Regional Facility, ID4.

Among health-qualified participants, a key skill was senior managers’ ability to share knowledge and build working relationships with other residential aged care facilities. Participants described how this helped design and implement processes that influenced the quality of residential aged care services. Interestingly, senior managers with a health qualification (but not those without) additionally stressed the importance of networking and collaboration with other organisations.

“As organisations, big or small, we [senior managers] need to collaborate and share our knowledge. We do not do that very well. We do have a regional facility management group meeting, and we talk. We talk to things that could help with raising quality within our facilities.” Clinical Operations Manager, Regional Facility, ID15.

As an important mechanism for developing positive residential aged care stakeholder relationships, almost all participants with a formal health qualification recommended that senior managers develop the skills to foster trust and rapport with residents and their families. The need for managers to effectively address resident complaints was strongly emphasised as a mechanism to ensure quality of care, as described by one participant:

“Your ability to manage complaints is important. It drives positive clinical care outcomes, and it helps to you to effectively negotiate with service providers to come in to look after your community in a way that the community expects.” Facility Manager, Regional Facility, ID7.

To sustain relationships with staff, participants with health qualifications noted that senior managers needed effective interpersonal skills such as active listening techniques to enhance teamwork across all levels of the organisation, as reported by this CEO, who possessed both nursing and business qualifications:

“The first thing that comes to mind is being able to listen. I think that is a key thing in terms of managing and caring for people. So, I am opened to listening to people. I've certainly learned to be more patient and take in what is happening around me before making a decision that could impact the way that care is carried out.” CEO, Regional Facility, ID1.

A manager’s ability to build and nurture collaborative relationships with residents, staff, and external providers was emphasised by both health-qualified and non-health qualified participants as important to promoting an organisation’s quality performance. As one qualified health practitioner reported:

“You have to understand people, relationships and what drives them. This helps you to pick up how you can get the best out of them. For the residents, you have to understand their story and what they need from you as the provider.” Clinical Care Coordinator, Rural Facility, ID3.

### Stewardship

‘Stewardship’ skills encapsulated the ability of senior managers to inspire organisational change and create a positive organisational culture that celebrates the diversity of staff and residents.

When considering the impact of organisational change, both senior managers with and without health qualifications spoke to the importance of interpreting industry regulations that influence health service delivery in the residential aged care sector, particularly during regulatory and legislative change. Among health-qualified managers, one CEO and one Facility Manager particularly emphasised the importance of senior managers having the skills and knowledge to comprehend and monitor legal and regulatory standards to influence quality of care.

“Above all, you need to be aware of and fully understand the frameworks and policies that dictate the way your organisation operates. Without this, you don’t know where to start when planning for quality compliance.” Facility Manager, Regional Facility, ID22.

“Managers need to be familiar with the legislation or the accreditation, all of the regulatory compliance issues that go with this unique type of industry [residential aged care].” CEO, Regional Facility, ID2.

One Director of Nursing further emphasised the importance of managers being able to interpret regulatory environments and then educate staff and residents regarding quality compliance to ensure quality performance across the organisation.

“Another really important skill is being able to educate patient care team members and the resident on the legislative and regulatory processes and methods for influencing both during daily operations.” Director of Nursing, Rural Facility, ID6.

Drawing attention to recent regulatory changes in Australia, including the introduction of the new Aged Care Quality Standards, several health-qualified participants reflected on the way senior managers needed the skills to serve as a ‘change agent’ to assist staff and residents in understanding reasons for change and to manage resistance to change effectively.

“Sometimes there are people who have worked in this industry for 30 or 40 years and [who] say this is the way they've always done it and they're not going to change, and so then your conversation has to be probably a bit more directive around, well, actually, it needs to change. You need to be a vessel to filter messages around change and make sure that actions follow.” Facility Manager, Rural Facility, ID19.

“So, my advice to a new manager is just very comfortable to listen, observe, sit back and understand, and get to know what you're actually dealing with. Without this insight, you will struggle to manage resistance to change.” Facility Manager, Regional Facility, ID8.

To minimise employee resistance, one Facility Manager with a tertiary Business qualification emphasised that skills to promote a collaborative approach to decision-making processes were necessary for empowering staff at all levels of the organisation to help embrace and champion change.

“I always aim to be motivating; a motivational leader that staff can follow and be inspired by, particularly when the message of change is on the table.” Facility Manager, Regional Facility, ID18.

The ability of senior managers to develop and lead a positive organisational culture was reported across both participant categories as a contributing factor to quality of care. Linking positive organisational culture to high-quality outcomes across the organisation, for example, one participant noted:

“Culture is critical, and something I always bang on about here is that you can walk into a workplace and within five minutes you can actually have a pretty good idea of what the quality of care would be like.” Facility Manager, Regional Facility, ID7.

The same Facility Manager also reported that the residential aged care leadership team should possess the skills to define diversity within its organisation.

“So, part of my role is I do some social profiling of our consumers and of our staff, trying to get to know who they are as a community. So, I definitely do try and orientate them to the different cultures and what's important. It helps everyone to feel connected to each other.” Facility Manager, Regional Facility, ID7.

### Professional development

‘Professional development’ skills identified by study participants included the ability of a senior manager to create working environments that promoted the accountability of internal and external services to the delivery of quality health care. Skills that were strongly emphasised by both participant categories within this domain included those of mentoring junior staff to participate in opportunities for continuing professional development and lifelong learning. Participants with formal health qualifications additionally emphasised skills relating to promoting staff accountability for residential aged care quality of performance.

“Managers need to devise strategies so that each department is accountable for the health care they provide. This way you are making everyone, regardless of their professional role, accountable for his or her actions. Regularly rewarding and showcasing high quality performance is really important.” Clinical Care Coordinator, Regional Facility, ID4.

To promote and sustain quality performance, health-qualified senior managers in this study emphasised the importance of skills to create and foster leadership teams to establish a professional work environment where both internal staff and external health care providers were responsible and accountable. A Clinical Care Coordinator operating in an inner regional residential aged care facility additionally noted that skills to design rewards, and positive feedback mechanisms, were an important part of effective leadership.

The capacity of senior managers to mentor junior staff and seek mentorship from respected colleagues was specified by both participant categories as an important residential aged care leadership skill.

“And I guess accessing mentoring is also important. Accessing other managers, who are really high performers and working ways to integrate this into your routine, can only help professional development.” Facility Manager, Rural Facility, ID7.

Of the qualified health practitioners interviewed, three Facility Managers reported that skills to mentor and coach junior managers to deliver sustained care quality within an organisation were important. Relatedly, participants with and without formal health qualifications described the self-awareness and opportunity of senior managers to actively seek mentorship from respected colleagues as an important skill within a residential aged care facility.

“Accessing mentoring was also important. Accessing other managers, and looking at, developing through them.” General Manager, Regional Facility, ID9.

### Knowledge of the healthcare environment

Skills linked to a manager’s understanding of competencies that influence qualitythe health care system and environment in which they operate were categorised under ‘Knowledge of the health care environment’ and were primarily referenced by participants who possessed a formal health qualification. Four participants, spanning different managerial roles and geographic areas of remoteness, suggested that senior managers require health knowledge to understand and interpret the scope of practice for the multiple and varied health care professions working within a residential aged care facility.

“Every manager should have basic clinical skills that you can continue to build on in whichever direction you need to through education and other avenues. Overall, you need to know what your staff are meant to be doing to ensure a safe, quality service.” Facility Manager, Rural Facility, ID 19.

One Director of Nursing also reported that senior managers required a developed knowledge and understanding of how to assess and observe clinical interventions.

“So, your clinical assessment and observation skills need to be really on the ball if quality is to prevail.” Director of Nursing, Regional Facility, ID17.

One Facility Manager mentioned the importance that senior managers have the knowledge to assess quality and safety performance, noting it was necessary to reward positive behaviours for staff who promote safe and quality health care practices.

“I always go back, and I complement them, and I do that often in front of the team at handover, saying this was really good in regard to your safety documentation. You were clear. So, my compliments are also very specific.” Clinical Care Coordinator, Regional Facility, ID4.

Of note, throughout most participant interviews, service quality was predominantly framed in relation to general business operations rather than quality of health care per se.

### Information technology and finance

Interviewees across both participant categories recommended residential aged care senior managers develop the knowledge and skills to promote the use of IT platforms within their organisations. Strategies included residential management teams employing innovative IT to deliver staff education regarding resident quality outcomes. IT was also reported as an approach to support the successful integration of regulations, including the new Aged Care Standards. Two managers located in inner regional residential aged care facilities reported that education contributed to increased knowledge across the organisation and a greater opportunity for high-quality care to be achieved.

“Yeah so it's about having information systems. So, it's important to have a structure where we have a forum where they concentrate on different topics to enhance the skills and knowledge or out staff across multiple areas.” Facility Manager, Regional Facility, ID16.

The ability of a senior manager to encourage the use of IT platforms was also linked to increased residential aged care efficiency and accuracy with documentation and quality reporting requirements, as emphasised by one General Manager who did not possess a health qualification.

“Senior managers must be aware of IT that can support quality reporting and compliance in line with its unique organisational profile. Senior managers must have knowledge regarding the operation of RACF IT platforms.” Facility Manager, General Manager, Regional Facility, ID9.

Another participant who possessed a tertiary qualification in Business and management experience within the hospitality industry strongly emphasised the importance of recognising finance’s role in quality improvement programs. This participant reflected that senior managers should possess the skills to effectively oversee the residential aged care financial position and ensure that appropriate resources are available to support high quality care.

“If you want to see quality outcomes, you need to know how to budget for quality staff, technology and other resources. Quality health care costs money.” Facility Manager, General Manager, Regional Facility, ID18.

## Discussion

Drawing on interviews with 19 individuals across 14 facilities in northern Australia, this study brings new knowledge regarding the leadership skills that Australian residential aged care senior managers perceived to be critical for promoting quality of care in often challenging regional, rural, and remote facility settings. Five domains of skills were identified by participants, including i) communication and relationship management skills; ii) stewardship skills; iii) professional development skills; iv) knowledge of the health care environment; and v) information technology and finance skills.

Overall, we found that participants emphasised communication and relationship management skills. Participants noted that senior managers’ ability to develop and nurture stakeholder collaborations, particularly those with clients’ families, regulatory bodies, and external service providers, was essential given the challenging resourcing environment in which many facilities operated. Communication and relationship management skills were also essential for workforce recruitment and retention in the face of resource shortages. Although often focused on health services generally rather than residential aged care, other studies have similarly identified effective staff communication strategies as an important leadership skill. Focusing on rural settings, for example, Lehmann et al. (2005) and Moosavi et al. (2020) showed that staff communication strategies often improve levels of employee job satisfaction [30, 31]. Moreover, the authors described the formation of mutual manager-employee relationships to enhance teamwork and self-reported employee well-being levels within an organisation [32].

Relatedly, stewardship skills, such as the ability to competently interpret and translate the increasingly complex regulatory requirements of the Australian aged care sector into facility-level strategies and operations, were noted as important. These findings draw attention to the importance of understanding the unique regulatory requirements of the aged care sector, and align with what is known about the importance of stewardship skills in mainstream healthcare. In a study involving healthcare managers in Swedish hospitals, for example, Andreessen et al. (2016) suggest that senior managers play an important role in supporting organisational change and should possess the knowledge to inspire new approaches to quality care [33]. Moreover, health care leaders contribute to strategic directions through their participation in senior-level decision-making about patient flow and staffing, quality improvement activities, and continuous learning opportunities to improve overall care delivery [34].

Findings from the current study support the importance of residential aged care senior managers’ having adequate health knowledge to influence quality of clinical care, with a sub-set of participants reflecting on the importance of health knowledge for designing and overseeing efficient, effective and client-centred clinical care. Participants with formal health qualifications reported knowledge of health professional scope of practice as important for assessing and monitoring the quality performance of staff. Such findings align with previous research in mainstream settings showing effective clinical leadership is linked to various functions [35] such as the achievement of regulatory objectives and timely care delivery [36]. In both mainstream and residential aged care settings, clinical knowledge is important to a manager’s capacity to form and enact quality-improvement systems [37]. To monitor compliance within these systems; however, findings from the current study additionally pointed to the importance of leadership skills to engage with and promote contemporary IT platforms as a strategy to enhance the accuracy and efficiency of quality reporting.

Interestingly, we found differences in emphasis on leadership skills between health qualified, and non-health qualified participants. The importance of leadership skills in the area of change management and strategic planning, for example, were emphasised by those with tertiary business qualifications but not by those with health qualifications. Likewise, financial management skills, including a manager’s ability to recognise the role of finance in quality improvement programs, were only reported by those with a formal business education. Conversely, participants without formal health qualifications did not mention health and health environment knowledge as critical skills. Such findings may indicate how particular professional backgrounds equip individuals with some, but not all, skills required to deliver leadership across the spectrum of residential aged care operations. With growing awareness of the range of clinical and business-related leadership skills likely necessary in this domain [10, 13], these findings emphasise the need for further work to establish a framework of leadership competencies for aged care and better understand the differentiated professional development needs of those with health and non-health backgrounds [6].

Although findings from the current study provide an important first step in addressing the evidence gap relating to leadership skills required by senior management personnel in Australia’s residential aged care facilities, we recognise that they are not comprehensive. Due to resource constraints, this study was not able to include residential aged care facilities owned and managed by government. This is a potential limitation as the unique regulatory and funding structures influencing the senior management role in government organisations are not represented in the study findings. Also, the focus on high performing aged care facilities may have limited the scope of study findings. Future research, including lower-performing organisations may broaden the category of themes identified in the current study and further enhance public discussion of leadership skills that influence the quality of residential aged care across a broader range of organisations. We also acknowledge that selected participants were from residential aged care facilities in northern Queensland. No participants managed facilities in major cities (ASGS-1), and the inclusion of such localities may have allowed additional exploration and comparison of the leadership challenges to quality care across multiple areas of geographical remoteness, broadening the generalisability of study findings.

## Conclusion

To better understand the optimal skill–mix of managers who lead Australian aged care services, the Royal Commission into Aged Care Quality and Safety - Final Report recommends that aged care leadership accountabilities and professional development strategies be better defined. This recommendation includes that residential aged care senior managers possess professional qualifications or high-level experience in management roles while receiving the continuous learning required to positively influence the quality of residential aged care within an increasingly complex health care environment. Yet, understanding of the critical skills needed by senior managers in Australian residential aged care is limited. In this study, we aimed to reduce this evidence gap and explore senior managers’ perspectives about which leadership skills are required to deliver high quality care in regional, rural and remote area residential aged care facilities.

Our findings demonstrate that senior managers view communication and relationship management skills and the ability to strategically plan and manage change as critical to ensuring service quality. Participants with different professional qualifications often emphasised certain leadership skills but did not mention others. For example, only those with formal health qualifications linked health knowledge and clinical skills to increased quality of care. Building on these important findings, further research is required to explore leadership skills and quality of care in government-owned residential aged care facilities to enhance the generalisability of research findings in this area. With ongoing concerns and challenges to residential aged care quality of care, more work is needed to identify sector-specific professional development strategies that prepare residential aged care senior management personnel with the appropriate skills to positively lead quality care within an increasingly complex environment.

## Supplementary Information

**Additional file 1.**

## Data Availability

The datasets used and analysed during the current study are available from the corresponding author on reasonable request.
