# Supplementary material for: A qualitative study of senior management perspectives on the leadership skills required in regional and rural Australian residential aged care facilities
Source: BMC Health Serv Res. 2022 May 18;22:667. doi: 10.1186/s12913-022-08049-4 (PMC9115967; doi:10.1186/s12913-022-08049-4)
Supplement: Supplementary file 1 — Additional file 1. [file 12913_2022_8049_MOESM1_ESM.docx]

**Appendix 1. In-depth interview guide**

1. Thank you for agreeing to chat with me, could you introduce yourself and your role in this organisation?
2. How long have you been in the role, and how did you come to be in this position?
3. Can you describe the senior management structure in this organisation / facility?
4. How is your role different from other senior management positions in this organisation?
5. Among the senior management staff, whose role would you say is most concerned with ensuring quality of health care?
   - 1. Why? How
6. When you think about ensuring quality of health care in a home/facility like this, what quality of care aspects do you feel it’s important to consider?

ALLOW respondent to answer – consider probes below, but be careful *not* to lead.

- - 1. Effectiveness
    2. Safety
    3. Accessibility
    4. Equity
    5. Responsiveness
    6. Efficiency

1. Do you or your colleagues use any quality frameworks to evaluate quality?
2. What are the main strategies you, and your colleagues use to maintain or improve quality of health care in this facility?
3. Thinking specifically about this home/facility, what do you see as the challenges to maintaining and improving quality of health for residents?

ALLOW respondent to answer – use below as follow-up probes “*What about…”*

- - 1. Finances / business model
    2. Leadership (education/experience/tenure of senior management + board)
    3. Number of staff (staffing ratio)
    4. Staff capabilities (training)
    5. Space (infrastructure)
    6. Team work and work culture (internal)

1. [If it hasn’t come up already] What are the key pieces of policy or regulation that influence quality of care in aged care homes in this region?
2. Do you think these challenges are the same as those experienced in mainstream health services, such as a hospital, or are there differences?
3. Thinking back to when you started this role, do you think you were well prepared to handle the challenges associated with quality of care that we just discussed? (please explain)
4. If you could have had more preparation or professional development before taking on your leadership role, in what areas would you have liked to have been upskilled?
5. Considering the increased pressure on aged-care homes from population aging and complex-co-morbidities, what do you think are the key features or characteristics required of senior management team to protect and promote quality of care?
6. And, relatedly, what organizational and policy settings are needed to better protect and promote quality of care in homes/facilities like this?
